# PyDESeq2: a python package for bulk RNA-seq differential expression analysis

**DOI:** 10.1093/bioinformatics/btad547

**Published:** 2023-09-05

**Authors:** Boris Muzellec, Maria Teleńczuk, Vincent Cabeli, Mathieu Andreux

**Affiliations:** Owkin France, Paris, 75009, France; Owkin France, Paris, 75009, France; Owkin France, Paris, 75009, France; Owkin France, Paris, 75009, France

## Abstract

**Summary:**

We present PyDESeq2, a python implementation of the DESeq2 workflow for differential expression analysis on bulk RNA-seq data. This re-implementation yields similar, but not identical, results: it achieves higher model likelihood, allows speed improvements on large datasets, as shown in experiments on TCGA data, and can be more easily interfaced with modern python-based data science tools.

**Availability and Implementation:**

PyDESeq2 is released as an open-source software under the MIT license. The source code is available on GitHub at https://github.com/owkin/PyDESeq2 and documented at https://pydeseq2.readthedocs.io. PyDESeq2 is part of the scverse ecosystem.

## 1 Introduction

Bulk RNA sequencing (RNA-seq) is one of the most common molecular data modalities used in biomedical research. Most RNA-seq datasets are used primarily for differential expression analysis (DEA) (Stark *et al.*, 2019), which provides invaluable insight on the associations between the genes’ expression and a phenotype. Due to the inherent noise and statistical challenges present in RNA-seq data, DEA methods have become more sophisticated over the past decade, making them difficult to re-implement or port over to new programming languages. In practice, the community now relies primarily on a handful of packages implementing state-of-the-art methods, among which DESeq2 (Love *et al.*, 2014).

While bioinformatics software is classically developed in R, a recent trend has seen the arrival of python software. Examples include the scanpy suite (Wolf *et al.*, 2018) or the squidpy package (Palla *et al.*, 2022) for single-cell and spatial RNA-seq, both of which are part of the scverse (Virshup *et al.*, 2023), an ecosystem of interoperable python omics packages.

This shift is motivated by several advantages of the python language: (1) the possibility to rely on well-maintained and efficient scientific computing packages such as numPy and sciPy, (2) a greater interoperability with machine learning and data science frameworks and (3) the potential to reach a wider audience, as python is one of the most popular programming languages (see, e.g. https://pypl.github.io/). Yet, to the best of our knowledge, there is currently no available python-native package for DEA with generalized linear models on bulk RNA-seq data.

A workaround consists in relying on python-to-R bindings, i.e. calling R software and making back-and-forth data conversions from a python interface, using packages such as rpy2 (https://rpy2.github.io/). However, this approach raises several issues: (1) it requires the user to install and maintain packages both in python and in R, which is cumbersome, (2) it creates computational overhead, as data are being converted and passed from one framework to the other, and (3) it may lead to a loss of control for the user, as the options and subroutines of the original packages are only accessible through the binding layer.

In an effort to alleviate those issues and to benefit from the advantages offered by python-based software, we present PyDESeq2, a python implementation of the bulk RNA-seq DEA methodology introduced by Love *et al.* (2014) and implemented in the R package DESeq2 (PyDESeq2 and DESeq2 are developed by independent groups.).

## 2 Implementation

PyDESeq2 implements the DEA methodology of Love *et al.* (2014), which briefly consists in modeling raw counts using a negative binomial distribution. Dispersion parameters are first estimated independently for each gene by fitting a negative binomial generalized linear model (GLM), and then shrunk toward a global trend curve. In turn, dispersions are used to fit gene-wise log-fold changes (LFC) between cohorts and to perform Wald tests for differential expression.

### 2.1 Available features and code structure

In version 0.3.5, the features implemented in PyDESeq2 correspond to default DESeq2 settings. More precisely, it implements the variance-stabilizing transformation, DEA for single-factor and n-level multi-factor designs (with categorical factors) using Wald tests, and LFC shrinkage using the apeGLM prior (Zhu *et al.*, 2019). Similarly to DESeq2, PyDESeq2 is structured around two classes of objects: a DeseqDataSet class, handling data-modeling steps from normalization to LFC fitting, and a DeseqStats class for statistical tests and optional LFC shrinkage. To fit GLMs, we rely on the popular scipy (Virtanen *et al.*, 2020) and statsmodels (Seabold and Perktold, 2010) python packages.

PyDESeq2 is an scverse ecosystem package, and relies on the anndata data structure (Virshup *et al.*, 2021). Because of this, PyDESeq2 analyses can be easily imported to and from any scverse package.

### 2.2 Comparison with DESeq2 on TCGA datasets

**Figure 1.**
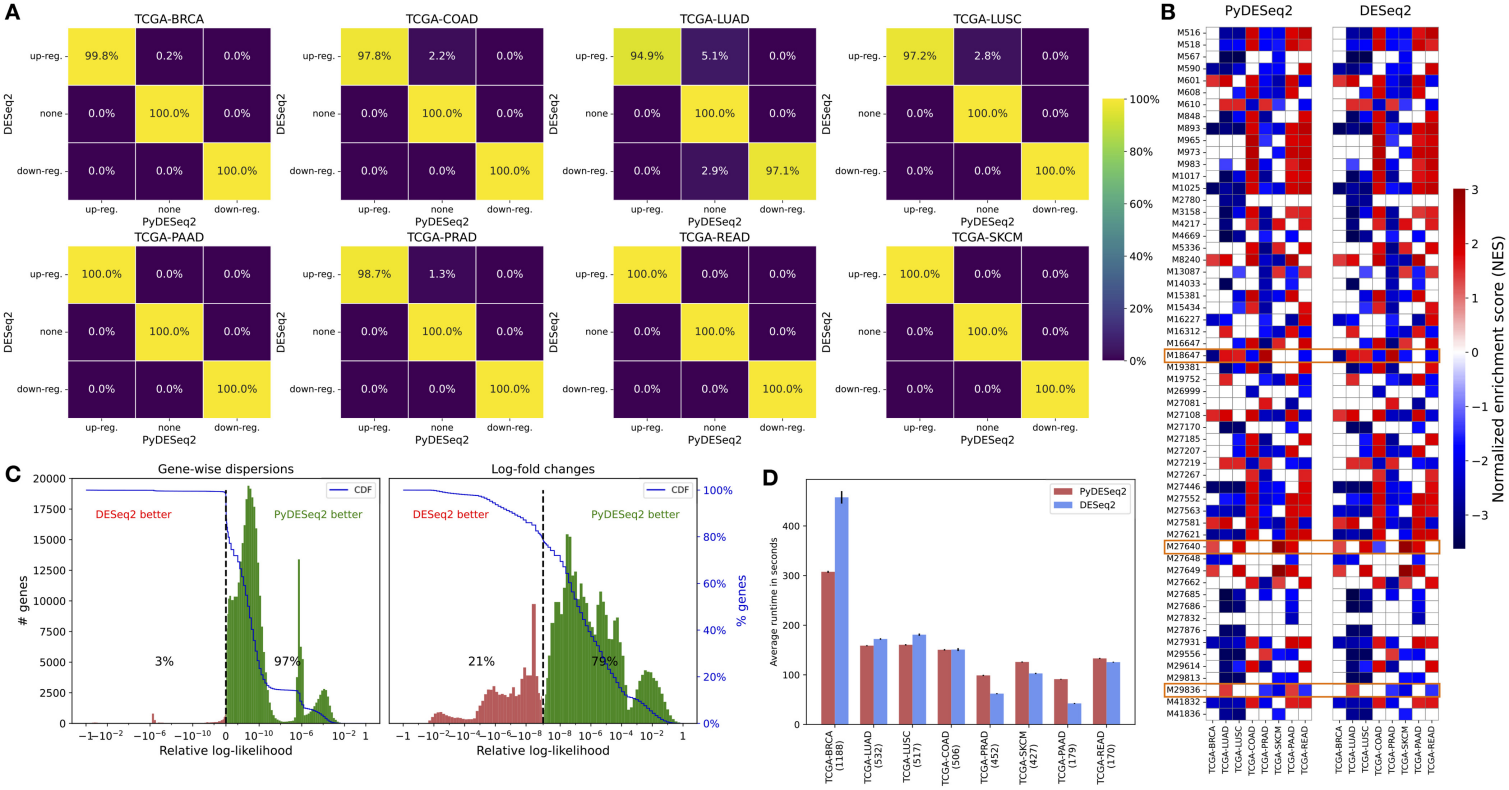
(A) Significantly differentially expressed genes (with padj≤0.05 and |LFC|≥2) according to PyDESeq2 and DESeq2. (B) Significantly enriched pathways (padj≤0.05) obtained with the fgsea package, using Wald statistics as gene-ranking metric. Only top 10 enriched pathways (according to adjusted *P*-value) of at least one cancer dataset are represented. If for a given cancer dataset, a pathway is not significantly enriched, the corresponding square is left blank. The three pathways which are considered significantly enriched in one implementation but not the other on a given TCGA dataset are highlighted by a surrounding box. (C) Distribution of relative log-likelihoods ($\frac{L(\mathrm{PyDESeq2})-L(\mathrm{DESeq2})}{\left|L(\mathrm{DESeq2})\right|}$), with corresponding cumulative distribution functions. (D) Time benchmark on an 8-core machine, averaged over 10 runs, using eight threads for each package. Numbers between parenthesis correspond to dataset sample sizes. (A–D) We refer to the Supplementary Appendix for additional details on the experiments.

In Fig. 1, we compare the results of PyDESeq2 and DESeq2 on eight bulk RNAseq datasets from The Cancer Genome Atlas (TCGA, https://www.cancer.gov/tcga). More precisely, we test differential expression between tissue samples corresponding to *non-advanced* vs. *advanced* tumor grades (as per TCGA’s clinical data), and focus on four criteria: retrieved genes, enriched pathways obtained with the fgsea package (Sergushichev, 2016), model likelihood, and speed.

As can be seen from Fig. 1, PyDESeq2 returns very similar sets of significant genes and pathways, while achieving higher model likelihood for dispersion and LFC parameters on a vast majority of genes, and at comparable speeds (higher for large cohorts, lower for small cohorts).

The data used in our experiments are publicly available on the TCGA website: https://portal.gdc.cancer.gov/. We refer to the Supplementary Material for additional details on the experiments.

### 2.3 Conclusion and future perspectives

In conclusion, PyDESeq2 is a fast and reliable package for bulk RNA-seq DEA. By releasing this package, we hope to fill a gap in the python omics ecosystem, and contribute to popularizing the usage of modern data science python tools in gene expression analysis.

Finally, let us mention some of the features that we plan to implement in PyDESeq2: future work includes adding support for continuous covariates, and likelihood-ratio tests.

## Supplementary Material

btad547_Supplementary_Data

## Data Availability

The data underlying this article were accessed from The Cancer Genome Atlas (https://portal.gdc.cancer.gov/).
