# Supplementary material for: PyDESeq2: a python package for bulk RNA-seq differential expression analysis
Source: Bioinformatics. 2023 Sep 5;39(9):btad547. doi: 10.1093/bioinformatics/btad547 (PMC10502239; doi:10.1093/bioinformatics/btad547)
Supplement: btad547_Supplementary_Data [file btad547_supplementary_data.zip › PyDESeq2_Bioinformatics_sup_mat.pdf]

A. Additional details on the experiments

A.1. PyDESeq2 and DESeq2 pipelines

For each dataset (downloadable from https://portal.gdc.cancer.gov/), we start with common row and column filtering steps. First, only samples whose tumor grade information (either *advanced* or *non-advanced*) are available are kept. Then, only genes with a total sum of read counts over those samples larger than 10 are considered in the study.

We used the default settings of both PyDESeq2 v0.3.5 (with python 3.8) and DESeq2 v1.40.2 (with R 4.3.0), corresponding to the "parametric" fitType for the latter. We refit Cooks outliers with a minimum number of replicates of 7, and performed an independent filtering step on the p-values to obtain ajdusted p-values with a threshold of 0.05 (note however that this threshold is not the default one in DESeq2). LFCs did not undergo a shrinking step.

In Fig. 1A, the genes are reported as significantly over (resp. under) expressed if the resulting adjusted p-value is under a 0.05 threshold, and if the corresponding log2-fold change is above (resp. under) a 2 threshold. Figures 3 to 10 display a detailed comparison of the retrieved genes for each dataset.

In Fig. 1C, all regularization terms involved in the objective functions that are optimized, such as Cox-Reid regularization and ridge penalties, are added to the model log-likelihoods.

Note that given that adjusted log-likelihoods are not necessarily concave or unimodal, some of the differences between PyDESeq2 and DESeq2 may be attributed to different starting points in the optimization.

A.2. Gene set enrichment analysis

For GSEA, the results of the PyDESeq2 and DESeq2 pipelines were processed using a common R script based on the fgsea package (Sergushichev, 2016). This script uses Wald statistics as a gene-ranking metric, and sorts pathways based on the computed adjusted p-value. Pathways with an adjusted p-value under 0.05 were considered significantly enriched.

Enrichment was tested for pathways from the C2:CP:REACTOME collection of the Molecular Signatures Database (MSigDB, http://www.gsea-msigdb.org/).

An index of all pathways appearing in Fig. 1B is available in Table 1.

Detailed GSEA plots for the 3 pathway/dataset combinations with significant differences visible in Fig. 1B are represented in Fig. 2.

A.3. Time benchmark

The time benchmarks summarized in Fig. 1D were run on a dedicated GCP instance of the n2-standard-8 type, with 8 virtual CPUs and 32GB of memory.

For each of the 8 datasets, the PyDESeq2 and DESeq2 pipelines were run using 8 cores, that is, setting n_cpus = 8 for PyDESeq2 and parallel = TRUE, BPPARAM = MulticoreParam(8) in DESeq2.

Each pipeline was run 10 times. Fig. 1D represents the resulting average time using solid bars, and the standard deviation with error bars.

A.4. Code

Scripts to reproduce the experiments are available in the supplementary material.

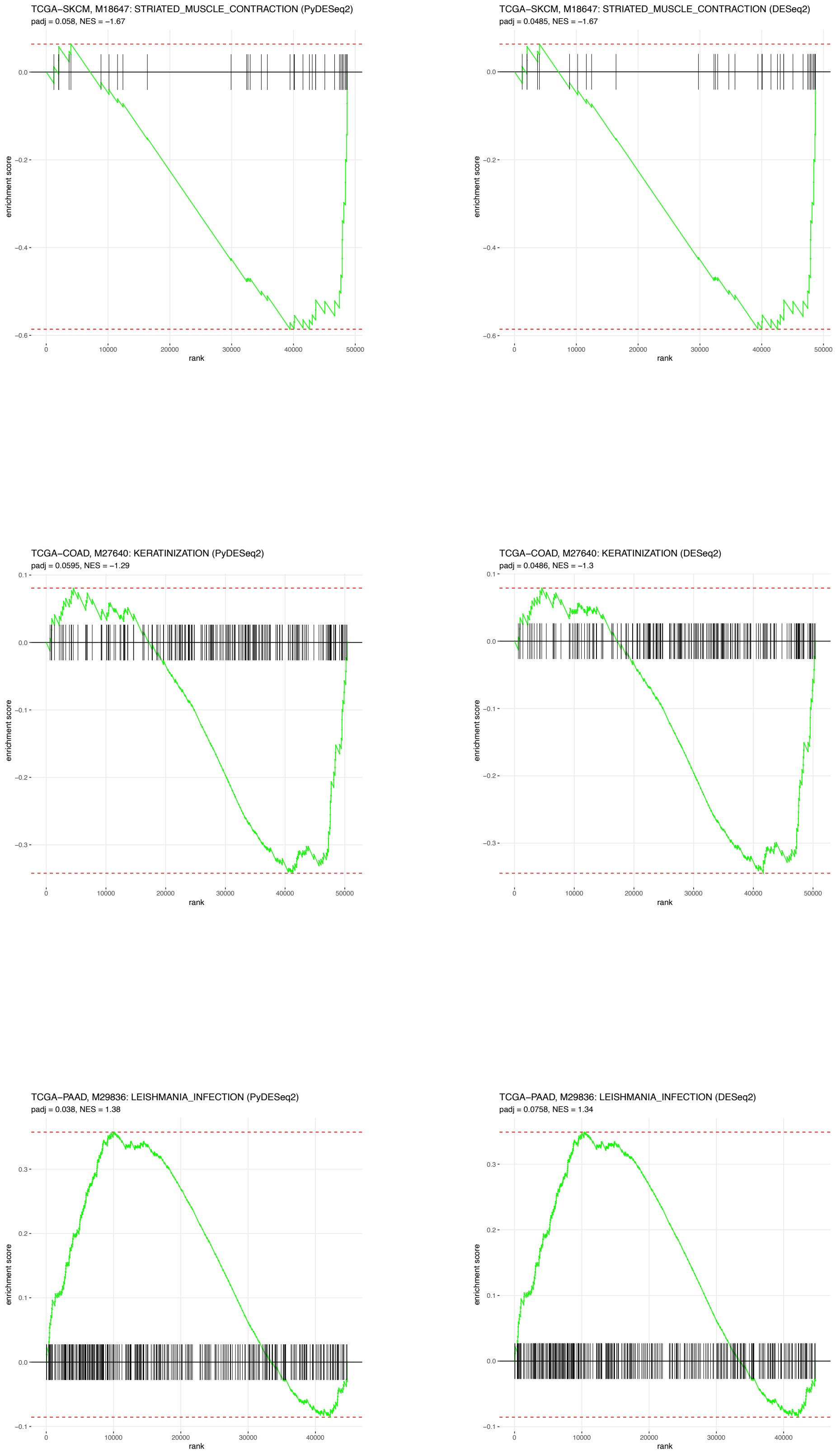

Fig. 2: Zoom on the 3 pathway/dataset combinations from Fig. 1B for which `PyDESeq2` and `DESeq2` significantly differ. As one can see, in all 3 cases the GSEA plots and enrichment scores are nearly identical, but the adjusted p-value falls short of the padj ≤ 0.05 for one implementation but not the other. This explains the differences observable in Fig. 1B (blank squares vs enriched pathways).

Table 1. Index of the pathways appearing in Fig. 1.

ID	Pathway
M516	THE CITRIC ACID TCA CYCLE AND RESPIRATORY ELECTRON TRANSPORT
M518	ANTIGEN PROCESSING CROSS PRESENTATION
M567	SRP DEPENDENT COTRANSLATIONAL PROTEIN TARGETING TO MEMBRANE
M590	MITOCHONDRIAL PROTEIN IMPORT
M601	ANTIGEN ACTIVATES B CELL RECEPTOR BCR LEADING TO GENERATION OF SECOND MESSENGERS
M608	SIGNALING BY THE B CELL RECEPTOR BCR
M610	EXTRACELLULAR MATRIX ORGANIZATION
M848	MITOTIC G1 PHASE AND G1 S TRANSITION
M893	RESPIRATORY ELECTRON TRANSPORT
M965	INTERFERON GAMMA SIGNALING
M973	INTERFERON ALPHA BETA SIGNALING
M983	INTERFERON SIGNALING
M1017	DNA REPLICATION
M1025	RESPIRATORY ELECTRON TRANSPORT ATP SYNTHESIS BY CHEMIOSMOTIC COUPLING AND HEAT PRODUCTION BY UNCOUPLING PROTEINS
M2780	SIGNALING BY ROBO RECEPTORS
M3158	S PHASE
M4217	MITOTIC PROMETAPHASE
M4669	INFLUENZA INFECTION
M5336	CELL CYCLE MITOTIC
M8240	IMMUNOREGULATORY INTERACTIONS BETWEEN A LYMPHOID AND A NON LYMPHOID CELL
M13087	PROCESSING OF CAPPED INTRON CONTAINING PRE MRNA
M14033	MRNA SPLICING
M15381	TCR SIGNALING
M15434	DNA REPAIR
M16227	CHOLESTEROL BIOSYNTHESIS
M16312	CELL SURFACE INTERACTIONS AT THE VASCULAR WALL
M16647	CELL CYCLE CHECKPOINTS
M18647	STRIATED MUSCLE CONTRACTION
M19381	G2 M CHECKPOINTS
M19752	COMPLEMENT CASCADE
M26999	COLLAGEN BIOSYNTHESIS AND MODIFYING ENZYMES
M27081	METABOLISM OF STEROID HORMONES
M27108	FCGR ACTIVATION
M27110	ROLE OF PHOSPHOLIPIDS IN PHAGOCYTOSIS
M27170	SELENOAMINO ACID METABOLISM
M27185	MITOTIC METAPHASE AND ANAPHASE
M27207	FCERI MEDIATED NF KB ACTIVATION
M27219	ECM PROTEOGLYCANS
M27267	TRANSCRIPTIONAL REGULATION BY TP53
M27446	MITOCHONDRIAL TRANSLATION
M27552	TNFR2 NON CANONICAL NF KB PATHWAY
M27563	DEFECTIVE CFTR CAUSES CYSTIC FIBROSIS
M27581	CD22 MEDIATED BCR REGULATION
M27621	COMPLEX I BIOGENESIS
M27640	KERATINIZATION
M27648	COPI MEDIATED ANTEROGRADE TRANSPORT
M27649	FORMATION OF THE CORNIFIED ENVELOPE
M27662	M PHASE
M27685	RRNA PROCESSING
M27686	EUKARYOTIC TRANSLATION INITIATION
M27832	METABOLISM OF STEROIDS
M27876	REGULATION OF EXPRESSION OF SLITS AND ROBOS
M27931	NEGATIVE REGULATION OF NOTCH4 SIGNALING
M29556	EUKARYOTIC TRANSLATION ELONGATION
M29614	SEPARATION OF SISTER CHROMATIDS
M29813	RESPONSE OF EIF2AK4 GCN2 TO AMINO ACID DEFICIENCY
M29836	LEISHMANIA INFECTION
M41832	REGULATION OF HMOX1 EXPRESSION AND ACTIVITY
M41836	CELLULAR RESPONSE TO STARVATION

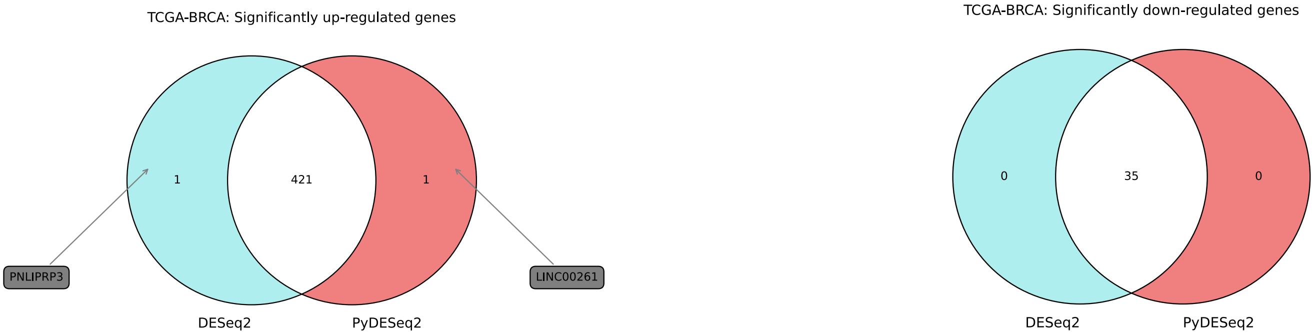

Fig. 3: TCGA-BRCA: `PyDESeq2` and `DESeq2` retrieved significantly differentially expressed genes (padj ≤ 0.05 and $|\text{LFC}| \geq 2$).

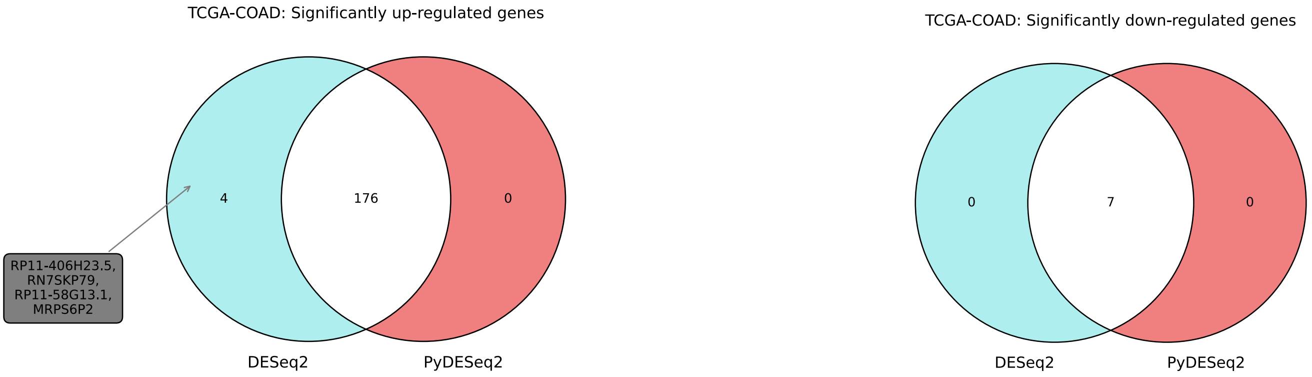

Fig. 4: TCGA-COAD: `PyDESeq2` and `DESeq2` retrieved significantly differentially expressed genes (padj ≤ 0.05 and $|\text{LFC}| \geq 2$).

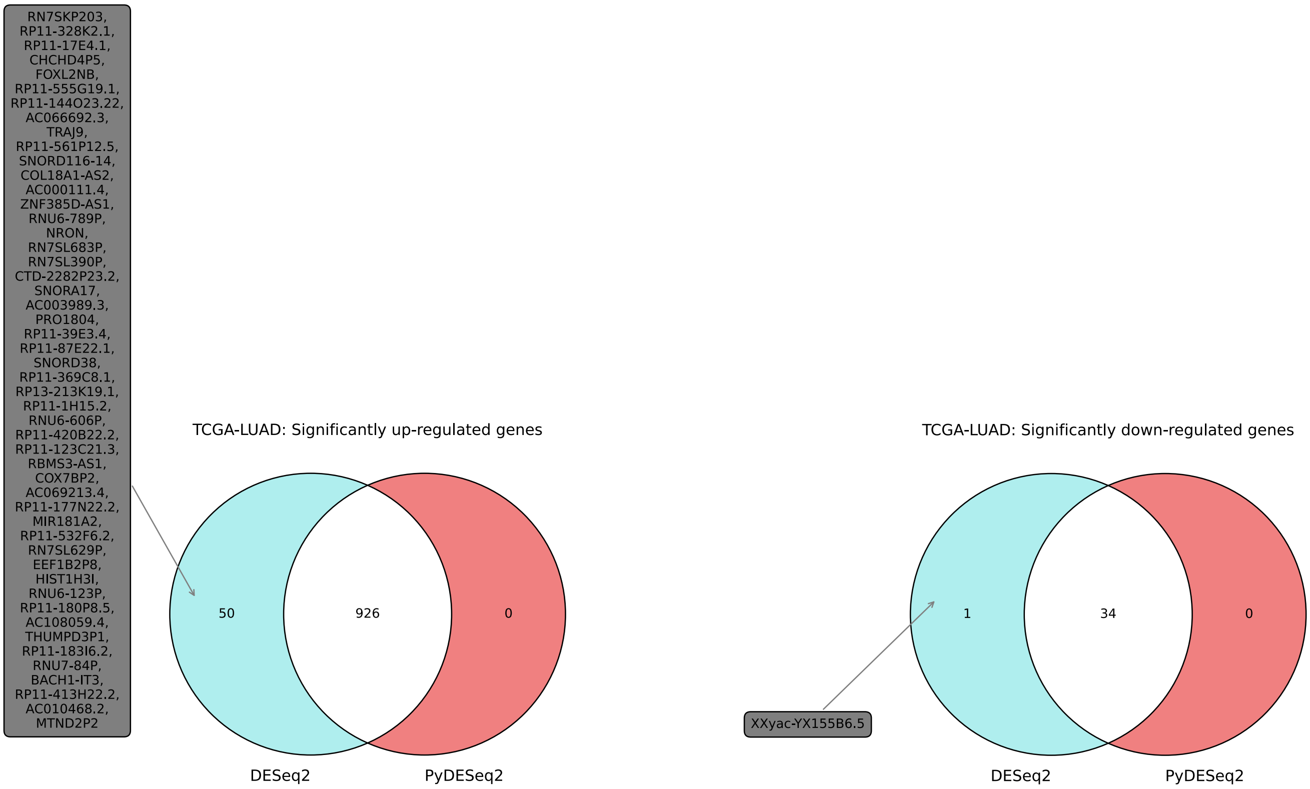

Fig. 5: TCGA-LUAD: `PyDESeq2` and `DESeq2` retrieved significantly differentially expressed genes (padj ≤ 0.05 and $|\text{LFC}| \geq 2$).

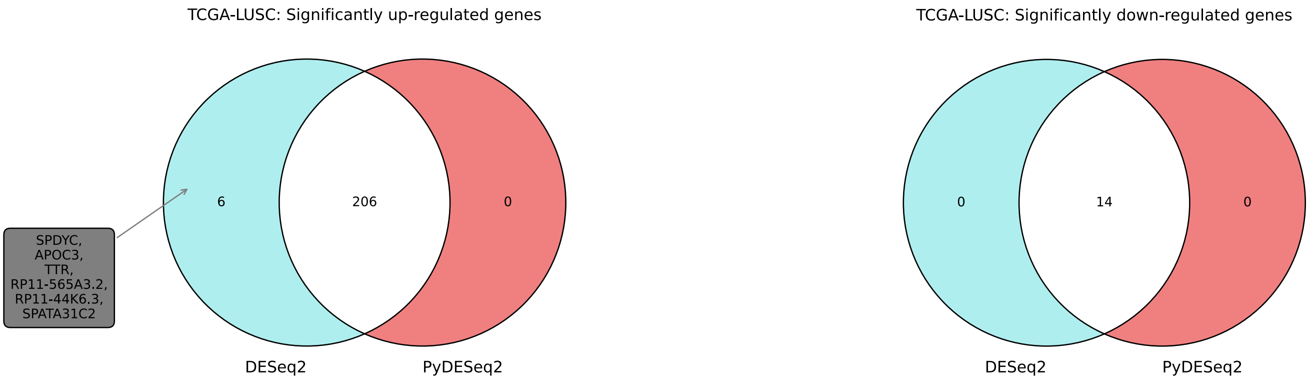

Fig. 6: TCGA-LUSC: `PyDESeq2` and `DESeq2` retrieved significantly differentially expressed genes (padj ≤ 0.05 and $|\text{LFC}| \geq 2$).

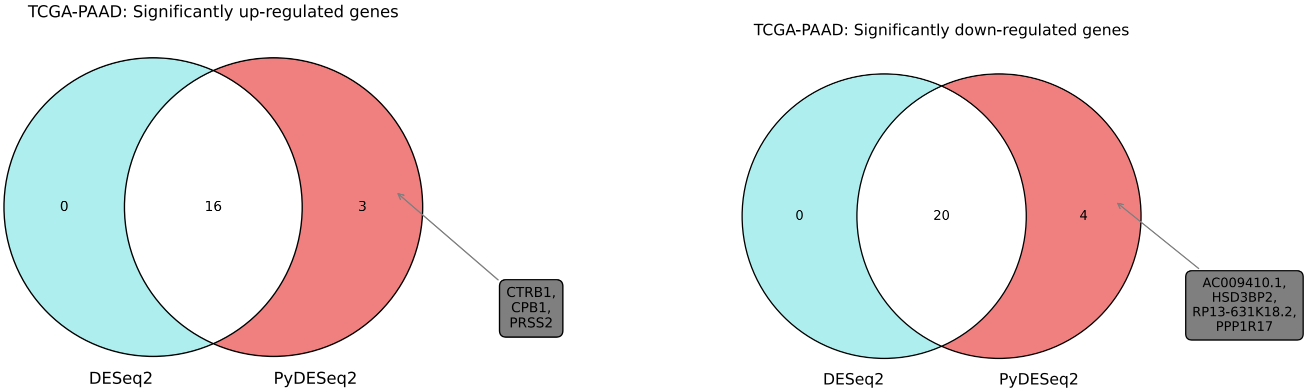

Fig. 7: TCGA-PAAD: `PyDESeq2` and `DESeq2` retrieved significantly differentially expressed genes (padj ≤ 0.05 and $|\text{LFC}| \geq 2$).

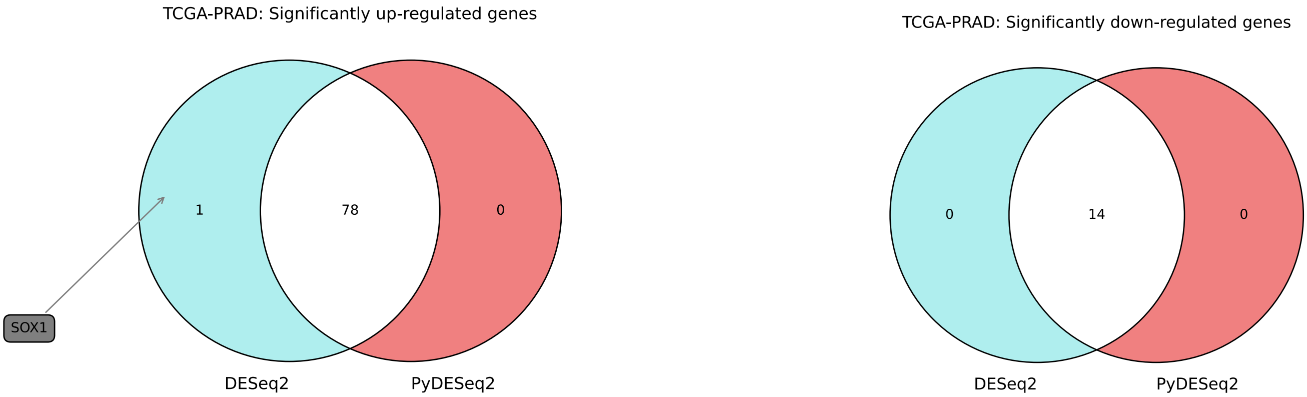

Fig. 8: TCGA-PRAD: `PyDESeq2` and `DESeq2` retrieved significantly differentially expressed genes (padj ≤ 0.05 and $|\text{LFC}| \geq 2$).

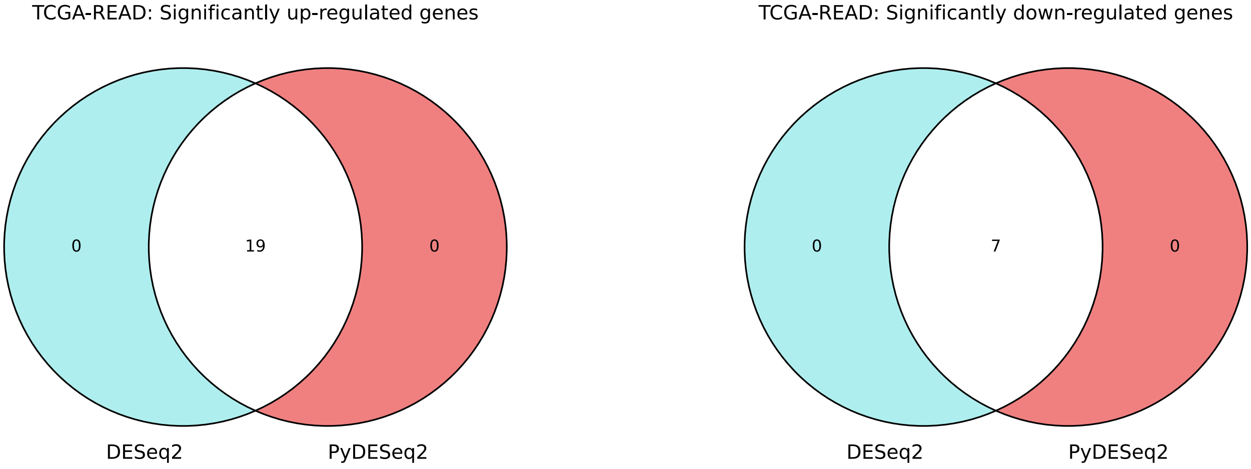

Fig. 9: TCGA-READ: `PyDESeq2` and `DESeq2` retrieved significantly differentially expressed genes (padj ≤ 0.05 and $|\text{LFC}| \geq 2$).

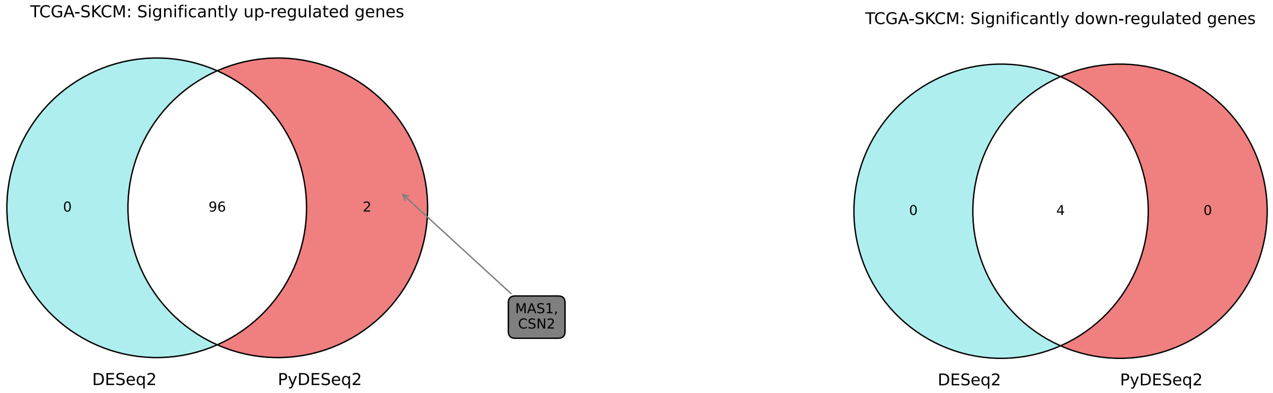

Fig. 10: TCGA-SKCM: `PyDESeq2` and `DESeq2` retrieved significantly differentially expressed genes (padj ≤ 0.05 and $|\text{LFC}| \geq 2$).
